# Liquid Biopsy is Instrumental for 3PM Dimensional Solutions in Cancer Management

**DOI:** 10.3390/jcm9092749

**Published:** 2020-08-25

**Authors:** Alena Liskova, Marek Samec, Lenka Koklesova, Frank A. Giordano, Peter Kubatka, Olga Golubnitschaja

**Affiliations:** 1 Department of Obstetrics and Gynecology, Jessenius Faculty of Medicine, Comenius University in Bratislava, 036 01 Martin, Slovakia; alenka.liskova@gmail.com (A.L.); marek.samec@gmail.com (M.S.); koklesova.lenka@gmail.com (L.K.); 2 Department of Radiation Oncology, University Hospital Bonn, Rheinische Friedrich-Wilhelms-Universität Bonn, 53127 Bonn, Germany; frank.giordano@ukbonn.de; 3 Department of Medical Biology, Jessenius Faculty of Medicine, Comenius University in Bratislava, 036 01 Martin, Slovakia; peter.kubatka@uniba.sk; 4 Predictive, Preventive and Personalised (3P) Medicine, Department of Radiation Oncology, University Hospital Bonn, Rheinische Friedrich-Wilhelms-Universität Bonn, 53127 Bonn, Germany

**Keywords:** cancer, predictive preventive personalized medicine (PPPM/3PM), liquid biopsy, blood, urine, saliva, tear fluid, cell-free circulating nucleic acids, circulating tumor cells (CTC), test sensitivity specificity

## Abstract

One in every four deaths is due to cancer in Europe. In view of its increasing incidence, cancer became the leading cause of death and disease burden in Denmark, France, the Netherlands, and the UK. Without essential improvements in cancer prevention, an additional 775,000 cases of annual incidence have been prognosed until 2040. Between 1995 and 2018, the direct costs of cancer doubled from EUR 52 billion to EUR 103 billion in Europe, and per capita health spending on cancer increased by 86% from EUR 105 to EUR 195 in general, whereby Austria, Germany, Switzerland, Benelux, and France spend the most on cancer care compared to other European countries. In view of the consequent severe socio-economic burden on society, the paradigm change from a reactive to a predictive, preventive, and personalized medical approach in the overall cancer management is essential. Concepts of predictive, preventive, and personalized medicine (3PM) demonstrate a great potential to revise the above presented trends and to implement cost-effective healthcare that benefits the patient and society as a whole. At any stage, application of early and predictive diagnostics, targeted prevention, and personalization of medical services are basic pillars making 3PM particularly attractive for the patients as well as ethical and cost-effective healthcare. Optimal 3PM approach requires novel instruments such as well-designed liquid biopsy application. This review article highlights current achievements and details liquid biopsy approaches specifically in cancer management. 3PM-relevant expert recommendations are provided.

## 1. Introduction

According to current statistics provided by the World Health Organization, one out of six deaths is due to cancer worldwide, demonstrating the annual mortality rate at a level of 10 million [1]. In Europe, even more than one in every four deaths (26%) is due to cancer. Cancer incidence increased by 50% from 2.1 million to 3.1 million cases between 1995 and 2018 in Europe. Due to increasing incidence, cancer became the leading cause of death and disease burden in Denmark, France, the Netherlands, and the UK. According to the comparator report on cancer in Europe 2019 (disease burden, costs and access to medicines), there will be an additional 775,000 cases of annual incidence until 2040 without improvements in cancer prevention [2].

The most common cancer types worldwide are lung (2.09 million cases), breast (2.09 million cases), colorectal (1.80 million cases), prostate (1.28 million cases), skin cancer (non-melanoma) (1.04 million cases), and stomach (1.03 million cases), whereas the most common causes of cancer-related death are malignancies of lung (1.76 million deaths), colorectum (862,000 deaths), stomach (783,000 deaths), liver (782,000 deaths), and breast (627,000 deaths). To this end, at least 30 to 50% of all cancer cases are preventable. In 2010, the total annual economic cost of cancers was estimated as USD 1.16 trillion [1]. Over the past decade, corresponding costs were permanently increasing. In Europe, the direct costs of cancer doubled from EUR 52 billion to EUR 103 billion between 1995 and 2018. Per capita health spending on cancer increased by 86% from EUR 105 to EUR 195, whereby Austria, Germany, Switzerland, Benelux, and France spend the most on cancer care compared to other European countries [2].

In view of the consequent severe socio-economic burden on society, the paradigm change from a reactive to a predictive preventive and personalized medical approach [3] in overall cancer management is essential [4]. The socio-economic burden can be illustrated by the example of prostate cancer as the disease for which the direct costs are increasing more quickly than those of any other cancer. As has been stated in “Prostate cancer management: long-term beliefs, epidemic developments in the early 21st century and predictive, preventive, and personalized medicine (3PM) dimensional solutions” (articles featured by the EPMA Journal, in July 2020), the implementing individualized patient profiles and adapted treatment algorithms would make the currently too heterogeneous landscape of prostate cancer treatment costs more transparent, providing clear “roadmap” for the cost saving. Further, due to systemic character of this multi-factorial disease, specifically liquid biopsy analysis is instrumental for disease prediction, prevention, and curative treatments at early stages [5]. To this end, circulating tumor cells (CTC) enumeration in liquid biopsy was highly prognostic at all steps of the disease-related analysis, being a more powerful indicator than other commonly used biomarkers [6]. In view of epidemics of non-communicable disorders characteristic of the early 21st century, liquid biopsy has become a key instrument in the context of 3PM [7,8]. This review article highlights current achievements and details liquid biopsy approaches specifically in cancer management. Data sources utilized biomedical literature available in the PubMed bibliographic database. Updated information published by scientific articles released in 2018–2020 has been given the highest priority. Traditionally strong is an application of biomarkers related to the blood-based biopsy. However, increasing evidence demonstrates that the application of complementary types of liquid biopsy is crucial for the overall quality of diagnostics [9,10,11,12,13,14,15,16]. The current article highlights very recent progress made in the area and provides prominent examples of a spectrum of liquid biopsy approaches with great potential to benefit several patient cohorts and to improve overall healthcare quality.

## 2. Liquid Biopsy Classification

Liquid biopsy is defined as an analysis of biomarkers in patients’ biofluids (non-solid tissue) such as blood, urine, cerebrospinal fluid (CSF), pericardial effusion, and others [17,18]. Liquid biopsy is non-invasive or minimally invasive, painless, precise, and real-time approach reducing the cost and diagnostic time with the advantage of overcoming the heterogeneity of tumors with the potential to replace tissue biopsy. Moreover, another advantage of liquid biopsy over traditional tissue biopsy is based on the need to monitor the tumor at separate times for an efficient treatment [6,19,20]. A big diagnostic advantage of liquid biopsy is that it can be performed in different body fluids [16]. The use of liquid biopsy in oncology is mainly concentrated on the analysis of circulating tumor cells (CTCs), circulating tumor nucleic acids (ctNA) or tumor derived extracellular vesicles (EV). Liquid biopsy is therefore beneficial for bringing new insight into the heterogeneity of cancer in terms of genetic and epigenetic alterations associated with disease development and response to treatment [17]. Liquid biopsy profiling based on epigenetic biomarkers has emerged as a minimally invasive and highly valuable approach of cancer management. Epigenetic modifications including DNA methylation, post-translational histone modifications or non-coding RNAs that are aberrantly deregulated in cancer are also markers applicable for liquid biopsy [21,22]. Moreover, the detection of additional multiple tumor biomarkers such as proteins, carbohydrates, lipids, metabolites or other cancer-derived substances allows for early cancer diagnosis, monitoring of the tumor evolution, and prediction of prognosis [23,24,25]. Despite intensive research on the methodology of liquid biopsy, the sensitivity as well as analytical specificity are still key obstacles of the development of new methods [26].

**Figure 1:**
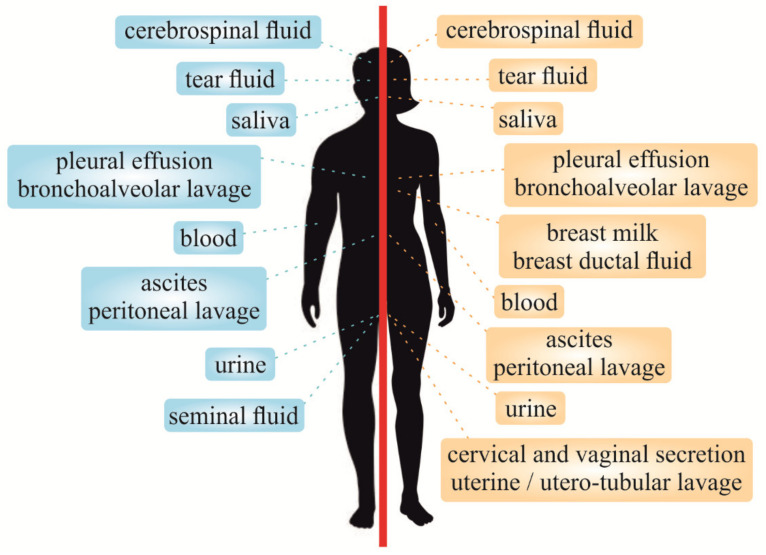
Analyses of the most prominent liquid biopsy types utilized for cancer-relevant biomarkers in males (left) and females (right).

Compared to tissue biopsy, liquid biopsy of blood samples can capture all subclones associated with the better view of cancer heterogeneity [27]. Although blood-based biopsy is well established, the exploration of urine, stool or saliva might contribute to better understanding of some malignancies [12]. Therefore, urine collection seems to be a promising alternative to blood-based biopsy, especially in specific types of cancer [28]. Liquid biopsy can be utilized also in other biofluids such as saliva, pleural effusions (PE), and CSF due to the content of tumor-derived genetic information, especially circulating cell-free tumor DNA (ctDNA) or other cancer-associated molecules [29]. Besides, malignant ascites as a result of peritoneal dissemination of cancer cells could be a good source of a liquid biopsy sample due to its accessibility, predominantly in gastrointestinal cancers [30]. Moreover, liquid biopsy concerning breast ductal fluid, breast milk, seminal fluid, cervical and vaginal secretions, and uterine/utero-tubular lavage can improve the management of various genital cancers of men as well as women [31,32,33,34,35,36]. Several other liquid biopsy samples including tear fluid, sweat, wound efflux, amniotic fluid or bronchoalveolar and peritoneal lavage can also provide benefits for the patient and healthcare at large [7,9,37,38]. Therefore, an increasing evidence shows that various types of liquid biopsy, illustrated in Figure 1, have the potential in cancer diagnostics, disease stratification, and monitoring of response to therapeutic interventions and could replace invasive tissue biopsy.

Despite great potential and many advantages over tissue biopsy, the utilization of liquid biopsy biomarkers is still associated with various challenges. High accuracy of biomarker detection within the scope of liquid biopsy is one of the most important challenges in clinical practice [39]. The lack of implementation of liquid biopsy into standard clinical practice, which is related to the unavailability of standardized techniques and guidelines, especially in terms of collection of samples and pre-analytical conditions, [26,40] can be illustrated by an example of cell-free DNA evaluation from blood and urine samples. The analysis of cell-free DNA from blood samples as a prognostic cancer biomarker is associated with various disparities among sample preparation, cell-free DNA isolation, and measurement of DNA concentration. Moreover, comparative or retrospective studies are impaired by the lack of generally accepted units for cell-free DNA quantification. In addition, the number of stabilizing reagents and dedicated blood collection tubes for the post-collection preservation of circulating markers in whole blood is growing [41,42] while the use of stabilization solution or ethylenediaminetetraacetic acid (EDTA) is highly recommended for plasma collection [26]. Furthermore, different extractions kits were demonstrated to produce a wide range of cell-free DNA yields ranging from 2.87 to 224 pg/μL. In addition, higher concentration of cell-free DNA has been observed in serum when compared with plasma in both healthy and cancer subjects [43,44]. While cell-free DNA released into blood circulation is between 116–161 bp long, urinary cell-free DNA is even more fragmented, ranging from 40–250 bp. Despite the fact that urine is gaining interest as a promising source of liquid biopsy due to its complete non-invasiveness, no standardized pre-analytical protocol of urine to preserve cell-free DNA is available. In fact, 32–75% of testing errors are associated with complications during the pre-analytical phase [40].

## 3. Liquid Biopsy Analysis: Advanced Technologies Manifest New Horizons in Cancer Management

As was described previously, liquid biopsy provides excellent opportunity for the detection of cancer-associated biomarkers based on DNA, RNA, proteins or other molecules obtained from various types of biofluids [45]. Cancer-associated biomarkers analyzed through liquid biopsy, including ctDNA, tumor-derived RNA (mainly microRNA), and CTCs, are dominant sources for further analysis via molecular techniques. Basic analytic methods (i.e., Sanger sequencing), which are widely used for the detection of DNA mutations extracted from tissue samples, are insufficient for the analysis of liquid biopsy samples due to their low sensitivity [29]. To overcome the limitation of the mentioned technique, massive progress in the field of molecular technologies has brought new opportunities for the clinical application of liquid biopsy. The potential of liquid biopsy for the diagnostic purposes to identify specific genetic or epigenetic changes is currently being tested in numerous studies using digital PCR, real-time PCR, and next-generation sequencing (NGS) [46].

### 3.1. Real-Time PCR

Real-time PCR (RT-PCR or qPCR) represents a relatively inexpensive and rapid method of amplification and measurement of the amount of DNA after each round of amplification cycle [47]. Many studies evaluated alterations in nucleic acids derived from biofluids using RT-PCR technology such as an analysis of aberrantly methylated *SEPT9* DNA in plasma of patients with colorectal cancer (CRC) [48]. Moreover, RT-PCR is a suitable method for the detection of micro RNA (miRNA) detected in plasma, CSF or exosomes of cancer patients [49,50,51,52]. In addition, urine samples also represent a promising source for a non-invasive biopsy that can be evaluated by quantitative PCR. As was demonstrated in bladder urothelial cell carcinoma patients, gene expression patterns of urine samples analyzed by TaqMan arrays can identify and predict tumor aggressiveness [53]. Furthermore, the quantification of urine-based cell-free miRNAs acts as an important biomarker monitoring cancer of the urogenital system in particular [54,55]. In addition, current research evaluating the use of liquid biopsy in the management of cancer highlights also saliva and PE as useful sources of miRNAs for RT-PCR [56,57,58,59].

### 3.2. Digital PCR

Absolute quantification of target nucleic acids can be evaluated via digital PCR (dPCR). Differences between RT-PCR and dPCR are in their strategy of evaluating the amount of amplificated products. The dPCR method includes droplet digital PCR (ddPCR) and BEAMing digital PCR based on beads, emulsion, amplification, and magnetics (BEAM). Importantly, ddPCR and BEAMing were used to detect mutations in ctDNA isolated from plasma or CSF of patients diagnosed with various cancer types including CRC, head and neck cancer (HNSCC), bladder cancer, malignant melanoma, central nervous system lymphoma or glioma [47,60,61,62,63,64,65,66,67,68,69]. Additionally, ddPCR was observed to be a promising technique for the analysis of minor amounts of DNA extracted from PE samples [70].

### 3.3. Next-Generation Sequencing

Next-generation sequencing (NGS) is a high throughput DNA sequencing methodology based on the parallel sequencing of several millions of short DNA sequences, which are subsequently alignment to reference genome or used for de novo sequence assembly [71,72]. Currently, NGS approaches play an important role in the understanding of genetic alterations associated with cancer. Importantly, the high sensitivity of ultra-deep NGS platforms allow their establishment as appropriate approaches in liquid biopsy analysis [73]. The ultra-deep NGS precisely identified a broad variety of oncogenic drivers and resistance mechanisms in non-small cell lung cancer (NSCLC) patients [74]. Moreover, CSF [75,76] and PE [77] represent another appropriate source of nucleic acid for NGS.

### 3.4. Proteomics Analyses of Liquid Biopsy

Circulating proteins represent promising markers for cancer diagnosis. To date, prostate-specific antigen (PSA), alpha-fetoprotein (AFP), CA-125, CA19-9, CA15-3, carcinoembryonic antigen (CEA), lactate dehydrogenase (LDH), HE4 or alanine and aspartate aminotransferases (ALT/AST) have been evaluated as cancer-associated biomarkers in many types of malignancies through liquid biopsy. Immune-based methods represent standard methodology for the analysis of the mentioned proteins. However, limitations of these methods include non-specific interaction of antibodies causing high false-positive rates or obstacles in technical issues such as a limited dynamic range of immunoassay [78,79]. Massive progress in proteomic technologies, including mass spectrometry (MS), opens new opportunities for the detection of cancer-associated biomarkers obtained via non-invasive liquid biopsy. Long et al. has recently used nanoporous silica coupled MALDI-TOF-MS to detect Bence-Jones protein from the urine of patients with multiple myeloma with high specificity and sensitivity [80]. Moreover, MALDI-TOF-MS based total serum protein fingerprinting has also shown high sensitivity and specificity for the diagnosis of liver cancer [81]. In addition, a combination of differential in-gel electrophoresis (DIGE) and MALDI-TOF-MS revealed efficacy in the identification of potential plasma biomarkers for esophageal squamous cell carcinoma [82].

We designed Table 1 for a detailed overview of the application of liquid biopsy through advanced technologies used in the selected studies of current oncological research.

## 4. Blood Samples as Currently Most Frequently Used Liquid Biopsy in Cancer Diagnostics

**Figure 2:**
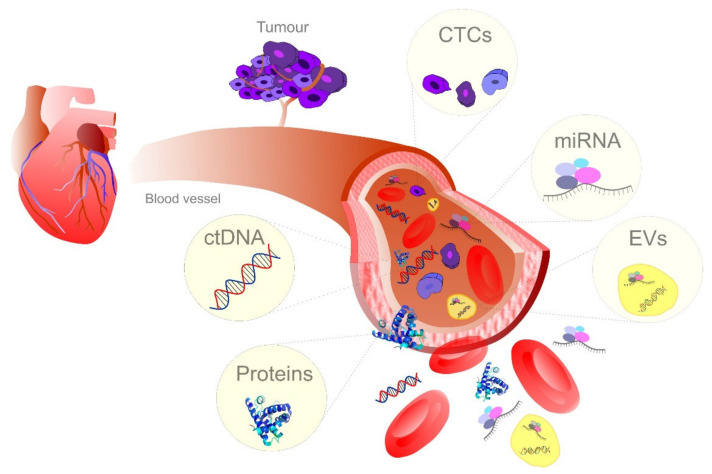
Blood-based cancer biomarkers in liquid biopsy. CTC, circulating tumor cells; ctDNA, Circulating: tumor cell-free DNA; EVs, extracellular vesicles.

The concentration of tumor-associated biomarkers released into the bloodstream might contribute to early detection of cancer and more favorable prediction of patients’ prognosis [84]. The emerging role of blood-based liquid biopsy in the management of cancer patients [85] in terms of predictive, personalized, and preventive medicine is based on the analysis of CTCs, genetic and epigenetic changes at the ctDNA level (such as mutations or DNA methylation) [86,87,88,89,90], EVs and their content derived from the original cancer cell [91,92,93], and protein-based markers [94,95,96,97] or their combinations [98] (Figure 2). Therefore, the detection of tumor biomarkers freely circulating in blood through liquid biopsy is associated with new opportunities for early diagnosis and therapy of cancer [99,100].

### 4.1. Circulating Tumor DNA

Circulating tumor cell-free DNA (ctDNA) is defined as tumor-derived extracellular nucleic acid in cell-free plasma or serum that is currently gaining a promising potential as a novel diagnostic and prognostic tool in cancer management [86,93,101]. Peripheral blood is the most common source of ctDNA but it could be detected also in other biofluids such as saliva, urine, ascites, and PE [102]. Optimal pre-analytical and analytical procedures are required for appropriate sample collection, centrifugation, storage, and DNA isolation from biological sources due to the contamination, low concentration, and high fragmentation of genomic DNA [42]. Despite the fact that a higher concentration of cell-free DNA is generally associated with serum, it is not a preferable liquid biopsy sample because of the contamination during the storage procedure [42,102] due to potentially high content of DNA released from lysis of circulating blood cells [103]. However, the comparison of the detection of the BRAF V600E-targetable mutation by dPCR from ctDNA and EV-derived DNA in plasma, serum, and CSF revealed serum to be more promising than plasma in liquid biopsy from pediatric central nervous tumors [39]. Above all, liquid biopsy profiling using ctDNA establishes an appropriate therapeutic approach, assessment of real-time conditions on cancer patients, and is associated with a potential of early detection of cancer [103]. The importance of liquid biopsy for cancer monitoring is associated with ctDNA that carries specific genetic and epigenetic aberrations related to cancer [104]. However, ctDNA proportion in total cell-free DNA varies due to the clinical-pathologic features of tumor and tumor microenvironment. Nevertheless, the level of ctDNA in standard blood is relatively low [105] as can be illustrated by plasma that constitutes <0.01–10% of total circulating cell-free DNA [103].

The concentrations of circulating ctDNA in serum represent a valuable tool in early diagnosis and prediction of prognosis of non-small cell lung cancer (NSCLC) patients [86]. An analysis of circulating cell-free DNA is a useful alternative for the selection of advanced NSCLC patients carrying activating mutations of *EGFR* who might benefit from the rechallenge with the first-generation tyrosine kinase inhibitor gefitinib [87]. Moreover, mutations in *PIK3CA* that are frequently observed in breast cancer could possess a predictive value for an evaluation of PI3K-selective inhibitor treatment. A concordance between *PI3KCA* mutations in tumor tissue and corresponding serum ctDNA from advanced breast cancer patients suggests an important role of the assessment of *PI3KCA* mutation status in the completion of imaging techniques as a marker of treatment response [106]. In addition, the detection of HPV-specific genes with the use of cell-free DNA in serum could serve as a marker for cervical cancer, estimating the risk of relapse [107]. Furthermore, combined isolation of ctDNA and exosomal RNA has been associated with increased sensitivity for the detection of EGFR mutations in the plasma of NSCLC patients [108].

The detection of DNA methylation represents one of the most promising approaches for cancer risk assessment [109]. Cancer-associated changes in DNA methylation can be identified in blood, stool, urine or other biofluids [110,111]. As was demonstrated by an example of breast cancer, blood-based approaches associated with cell-free DNA are minimally invasive screening tools used to define epigenetic alterations of genes serving as liquid biopsy, complementing conventional detection tools such as mammography [112]. Moreover, the detection of DNA methylation in plasma or serum samples may help to understand tumor heterogeneity better than tissue biopsy [89]. An evaluation of promoter methylation in a serum test, serum validation, and a plasma cohort revealed that the promising potential for early breast cancer detection could be attributed to the novel hypermethylation biomarker panel including *SPAG6*, *NKX2-6*, *ITIH5,* and *PER1* [112]. Additionally, the assessment of the methylation level of selected genes in circulating cell-free DNA in plasma samples may be considered an effective procedure for the subtyping of lung cancer in addition to standard diagnostic procedures [89]. Last but not least, more than fifty cancer types at metastatic and non-metastatic stages have been recently detected through cell-free DNA sequencing of informative methylation patterns, while its sensitivity and specificity approached the goal for population-level screening [113].

### 4.2. Circulating Cell-Free miRNAs

miRNAs are small non-coding RNAs consisting of 19–25 nucleotides that are importantly associated with cancer biology by regulating the expression of oncogenes or tumor suppressors at the post-transcriptional level. Circulating miRNAs are stable in plasma and serum for quantification due to the formation of complexes with proteins [114] and resistance to RNase activity, extreme pH, and freeze–thaw cycles [115], especially due to the incorporation within EVs (more details on miRNAs incorporated within EVs in the following section) [114]. Therefore, the detection of miRNA or a combination of multiple miRNAs in plasma or serum represents a potent circulating biomarker for cancer management [116,117].

The combination of miR-125a-5p, miR-25, and miR-126 has shown potential for the early detection of lung cancer demonstrated by a phase 2 study including a preliminary marker selection and a validation phase on serum samples [118]. Moreover, a combination of serum miR-17-3p and miR-1185-2-3p has been identified as a promising biomarker for the detection of prostate cancer, while high sensitivity and specificity of this model could improve the accuracy of the diagnosis [119]. Similarly, a panel of seven miRNAs (miR-6087, miR-1185-1-3p, miR-3960, miR-6724-5p, miR-1343-5p, miR-6831-5p, and miR-4695-5p) could be used as a serum biomarker for early detection of bladder cancer [120]. An evaluation of deregulated miRNAs could also improve the early detection of female malignancies. Several miRNAs (miR-518b, miR-4719, and miR-6757-3p) have been observed to be deregulated in breast cancer serum, and miR-484/-23a could serve as a potential diagnostic biomarker for endometrial and ovarian cancer [121].

### 4.3. Extracellular Vesicles in Liquid Biopsy

The evaluation of cancer biomarkers associated with extracellular vesicles (EVs) that are included in biological fluids is rapidly advancing possibility to be used in cancer management [122]. Multiple methods of EV isolation have been developed, including ultracentrifugation, size-exclusion chromatography, polymer-based isolation, immunoselection or novel methods such as affinity-based technique using Tim4 protein binding to phosphatidylserine, an enriched component of the EVs’ surface [123,124,125]. The importance of EVs in early-stage diagnosis of cancer, monitoring of the treatment, and precision therapy is based on their high abundance in biofluids, accessibility from liquid biopsy as well as the specific content from the cell of origin [126]. Therefore, EVs contain a composition of molecules such as lipids, proteins, metabolites or nucleic acids that are well protected due to a lipid bilayer membrane of EVs, even if extracted from circulating or proximal biofluids [38,87,116]. EVs including circulating exosomes have recently gained an increased importance due to the applicability in the liquid biopsy field and their potential as biomarkers for cancer screening and monitoring [91].

Circulating exosomes could serve as novel non-invasive biomarkers for the detection of esophageal squamous cell carcinoma (ESCC), as has been demonstrated by Zhao et al. in a study evaluating the serum samples of ESCC patients and that revealed that circulating exosomes are stable enough to be measured. Moreover, the serum concentration of circulating exosomes was significantly higher in ESCC patients when compared with healthy controls and could distinguish patients with ESCC with a sensitivity of 75% and a specificity of 85%, while an increased level of circulating exosomes also correlated with poor overall survival and progression-free survival [91]. Furthermore, circulating exosomal DNA could be used for fast and low-cost identification of cancer-driving mutations, as has been illustrated by a study evaluating a possible identification of *KRAS* and *TP53* mutations in patients with pancreas-associated pathologies, including pancreatic ductal adenocarcinoma, chronic pancreatitis, intraductal papillary mucinous neoplasm as well as in healthy subjects [92]. Additionally, EV-miRNAs are other indispensable cancer biomarkers of liquid biopsy [116] due to their high stability as a result of encapsulation within EVs. Serum EV-miRNA-21, -92a, and -222 have exerted great potential as liquid biopsy biomarkers for the diagnosis and prognosis of bevacizumab-treated metastatic colorectal cancer patients [93].

However, an isolation of EVs still represents a challenge given their nanoscale dimensions and low buoyant density. Nevertheless, nanoscale deterministic lateral displacement has been demonstrated as a promising technology for fast, automatable, and reproducible isolation of EVs [126]. Moreover, a far-field nanoplasmon-enhanced scattering (FF-nPES) has been recently described as an appropriate assay for isolation-free characterization of EVs in small volumes of serum. Eventually, FF-nPES could be used for the direct analysis of EV epithelial cell adhesion molecule (EpCAM) expression from serum in order to differentiate the early stage of pancreatic ductal adenocarcinoma patients from healthy controls [127]. Additionally, Lewis et al. have recently presented a method integrating capture and analysis of exosomes and other EVs from whole blood, plasma or serum onto an alternating current (AC) electrokinetic microarray chip with subsequent fast on-chip immunofluorescence analysis, allowing an identification and quantification of target biomarkers to detect pancreatic cancer in patient blood [128]. Importantly, an analysis of bloodborne nanoscale EVs (nsEVs) has been associated with a promising potential as a diagnostic test that could predict and monitor the effectiveness of treatment strategy and identify new targets in pathological conditions including cancer [129].

### 4.4. The Significance of CTCs in Blood-Based Liquid Biopsy for Cancer Management

CTCs are defined as cancer cells that shed off the tumor site and enter the lymphatic and circulatory system [130,131,132]. Apart from the role of single CTCs in the seeding and dissemination of cancer, CTC cluster-mediated metastasis has recently emerged as at least as important. The different features of CTC clusters in comparison with single CTCs are related to the phenotype, gene expression signature, and dissemination mode [133].

Due to their contribution to metastasis, CTCs belong to most widely studied markers in liquid biopsy [130,131] and show a great potential as diagnostic, prognostic, and predictive biomarkers in cancer management [20,134]. Both plasma and serum are insufficient samples for the purpose of the analysis of CTCs due to the removal of cellular structures during centrifugation. Therefore, whole blood is the most common biofluid used for liquid biopsy based on the evaluation of CTCs. However, several obstacles are related to the isolation, detection, and analysis of CTCs including their rarity (1–100 cells per milliliter of blood) and similarity in size to white blood cells [130]. Another disadvantage of CTC analysis is their heterogeneous nature demonstrated by significant variations in surface expression of biomarkers within various groups of CTCs [131]. The dynamic nature of surface marker expression is best illustrated by an example of CTCs undergoing epithelial mesenchymal transition [135]. Nevertheless, numerous technologies and platforms allowing the isolation and analysis of CTCs for the purpose of their potential use in the management of cancer patients are currently emerging [136].

The great potential of CTCs as cancer-associated biomarkers in blood-based liquid biopsy has been recently demonstrated by several authors. The study evaluating the value of changes in CTCs as an indicator of cancer progression with low pre-treatment CTCs has revealed that increasing CTCs during the first twelve weeks of chemotherapy or endocrine therapy is independently associated with worse overall survival of advanced prostate cancer patients [137]. Moreover, the determination of numbers of mesenchymal and epithelial CTCs could be utilized as a predictive marker of the survival of metastatic breast cancer patients receiving eribulin [138]. In addition, CTCs could be used as a biomarker of treatment efficacy with predictive and prognostic value as has been demonstrated by the ability of the tyrosine multikinase inhibitor pazopanib to eliminate different subpopulations of CTCs in patients with relapsed small-cell lung carcinoma (SCLC) [139]. Furthermore, CTCs have been detected in 90% of newly diagnosed epithelial ovarian cancer patients, and the number of CTCs correlated with the stage. Moreover, the level of CTCs changed with treatment, while the expression of EpCAM and human epidermal growth factor receptor 2 (HER2) in CTCs correlated with chemotherapy resistance [140].

The utilization of CTCs in liquid biopsy is associated with the need for necessary progress in the methods or technologies enabling improved identification or isolation of CTCs and subsequent analysis applicable in the management of cancer patients. Based on preceding screening of EGFR and HER3 expression in primary and metastatic NSCLC tumor tissue, the CTC detection method based on these two markers has been developed and exerted the possibility to capture a larger fraction of CTCs also in combination with EpCAM in NSCLC patients. Eventually, an isolation of CTCs based on multiple markers may provide important knowledge of cancer biology and treatment through the novel liquid biopsy approach [141]. Moreover, chemosensitivity assays using liquid biopsy-derived CTCs from metastatic epithelial ovarian cancer patients in progression after systemic chemotherapy have been demonstrated to be feasible and useful to predict response to treatment [142]. Furthermore, Benin et al. have recently demonstrated that CTCs in peripheral blood of Ewing sarcoma patients could be identified by immunoseparation with CD99 antibody and magnetic microbeads, suggesting prognostic and predictive significance of this method as a liquid biopsy approach. Above all, the isolation of CD99-positive CTCs could improve the understanding of the metastatic precursor subpopulation and clinical-decision making [143]. In addition, the detection of CTCs in drainage venous blood using a new filtration and cytology-based automated platform provides a unique prognostic and diagnostic tool of liquid biopsy in CRC patients [144].

Above all, the utilization of CTCs in liquid biopsy is associated with great potential for the management of cancer patients, including the detection of disease, monitoring of metastasis, and personalized anti-cancer strategies. However, further progress in the research focusing on methods of CTC isolation, detachment and detection with increased specificity and sensitivity would allow the translation of fundamental research into clinical application. The study of CTCs offers opportunities for their application in liquid biopsy and development of new cancer biomarkers for the diagnosis, prognosis, prediction, and monitoring of the disease and response to therapy [131,136].

### 4.5. Other Biomarkers of Blood-Based Liquid Biopsy or Their Combinations

The level of soluble forms of programmed death receptor-1 and -2 (sPD-L1 and sPD-L2) in liquid biopsy of serum samples has been highlighted as a complementary biomarker that reflects clinical status, response to treatment, and disease outcome of epithelial ovarian cancer. Moreover, serum PD-L1 in particular could facilitate the identification of high-risk patients with poor disease outcome despite platinum-sensitivity arguing for additional therapy [145]. Additionally, serum calretinin, a calcium-binding protein controlling intracellular calcium signaling, is suggested as liquid biopsy marker that could be used for an independent prediction of platinum resistance and prognosis in ovarian cancer [94]. Similarly, serum gastrokine 1 (sGKN1) could be used as a highly specific biomarker for the detection of early and advanced gastric carcinoma [95]. Additionally, Du et al. have indicated that an analysis of a combination of squamous cell carcinoma antigen (SCC Ag) degree and miRNA-29a, miRNA-25, miRNA-486-5p levels in serum could improve the detection of early-stage cervical cancer [98]. Moreover, an evaluation of serum signature of six miRNAs (miR-15b-5p, miR-29a-3p, miR-335-5p, miR-18a-5p, miR-19a-3p, and miR-19b-3p) and fecal hemoglobin concentration has revealed high accuracy of the detection of advanced colorectal neoplasia in average-risk individuals [146].

Aberrant regulation of post-translational histone modifications is importantly associated with processes of carcinogenesis that could be used as a biomarker or potential target for cancer therapy. Liquid biopsy allows non-invasive analysis of histone modifications and their modifiers that could potentially improve the management of cancer patients, including sub-grouping of them for epi-drug treatment, predicting response to therapy, relapse or prognosis, as was demonstrated by a study of hepatocellular carcinoma. The serum purified histones of hepatocellular cancer patients have shown a comparable pattern of modifications such as methylation (H4K20Me3, H3K27Me3, H3K9Me3), phosphorylation (γ-H2AX and H3S10P), and acetylation (H4K16Ac) to paired cancer tissues [96]. Moreover, an evaluation of the potential of detection of CRC by histone methylation marks in plasma has revealed significantly lower H3K27me3 and H4K20me3 in CRC patients when compared to healthy controls [97].

An identification and validation of serum biomarker signature-based liquid biopsy in two large case–control studies of patients with pancreatic ductal adenocarcinoma (PDAC) have shown the ability to detect samples from patients with stage I and II PDAC with high accuracy. Therefore, if a proteomic multiparametric analysis discriminating patients with early-stage I and II from controls is supported by prospective validation studies, it could gain clinical importance in the surveillance of high-risk patients (hereditary PDAC, chronic pancreatitis, Peutz–Jeghers syndrome), patients with late-onset diabetes who are at higher risk of acquiring PDAC and patients with vague abdominal symptoms [147]. Moreover, a differential scanning calorimetry thermogram analysis of a serum sample termed thermal liquid biopsy (TLB) by Rodrigo et al. is a valuable approach for personalized diagnostic assessment of cancer patients. The prediction score of TLB is based on the detection of plasma/serum proteome alterations through calorimetric thermograms that correlates with the presence of lung cancer (91% accuracy rate, 90% sensitivity, and 92% specificity). Above all, TLB could be applied in clinical practice for the screening and monitoring of lung cancer, while its advantages include quickness, minimal invasiveness, and low risk [148]. Table 2 shows a detailed overview of current research focusing on the potential use of blood-based liquid biopsy in precise and personalized strategies targeting malignant disease in the 21st century.

## 5. Other Liquid Biopsy Types

Apart from blood, promising results of liquid biopsy have been attributed to other biofluids such as urine, saliva, CSF, ascites, PE, cervical and vaginal secretions, tear fluid, breast milk, breast ductal fluid, and seminal fluid, as well as bronchoalveolar, peritoneal or uterine/utero-tubular lavage [26,35,76,107,149,150,151,152,153,154]. The potential use of liquid biopsy is discussed also in terms of pediatric cancers. Despite the fact that blood might be the first step in liquid biopsy diagnostics in the future, it could be also complemented/replaced by other body fluids such as urine, saliva, or CSF [14].

### 5.1. Urine-Based Liquid Biopsy

When discussing the great potential of 3PM medicine in the current progress associated with cancer management [8,155], urine as a source of important biomarkers of liquid biopsy should also be precisely evaluated. The collection of urine is completely non-invasive and therefore could serve as an alternative biofluid source of liquid biopsy markers [28]. Due to invasive surgical procedure required for the tumor tissue biopsy of a genitourinary system, urine can prove to be “liquid gold” [156]. In addition, urine-based liquid biopsy could have great potential in non-urological cancer screening, detection of cancer, and monitoring of recurrence and metastasis [28].

The measurement of cell-free DNA in urine is an ultra-non-invasive tool carrying genetic information from cells shedding into urine or transporting from circulation [157]. Screening from serial urine supernatants has revealed an association between high levels of tumor DNA and later stages of non-muscle-invasive bladder cancer [158]. Moreover, a possible use of urine-based liquid biopsy for the detection and clinical staging of prostate cancer patients demonstrated by gene promoter methylation assay has been related to the number of genes methylated (NGM) value for a six-gene panel (*APC2*, *CDH1*, *FOXP1*, *LRRC3B*, *WNT7A,* and *ZIC4*) [159]. In addition, both plasma and urine ctDNA could be utilized as markers of colorectal cancer progression [160]. Similarly, the detection of *EGFR* and *TP53* mutations through the combination of plasma, urine, and sputum could improve the predictive value of personalized treatment of advanced NSCLC [161]. However, sensitive and accurate methods allowing high depth of sequencing and minimization of artifacts are required for the molecular detection of mutations in urinary DNA. Nevertheless, sequencing methods such as Tagged amplicon deep sequencing (TAm-Seq), Safe-sequencing system (Safe-SeqS), Fast Aneuploidy Screening Test-Sequencing System (FAST-SeqS), and CAncer Personalized Profiling by deep Sequencing (CAPP-Seq) to overcome this disadvantage have been developed [156].

The utility of urinary miRNAs as important cancer biomarkers is supported by their relative stability under various storage conditions [162]. Regarding clear cell renal cell carcinoma, urinary cell-free miR-210 could be used as a tool for the diagnosis of the disease [163]. In addition, the urine-based detection of selected miRNAs that are altered in different bladder cancer subtypes could be an accurate diagnostic tool of its early diagnosis [164]. The potential importance of urine-based liquid biopsy in the management of cancer also includes screening of urine-derived exosomes for miRNAs in endometrial cancer patients [165].

Additionally, molecular lipid species in urinary exosomes have also exhibited potential utility as prostate cancer biomarkers [24].

Therefore, complementary to blood, other body fluids such as urine demonstrate great potential to significantly improve overall diagnostic quality [13]. A detailed description of the selected studies to assess current knowledge on urine-derived liquid biopsy in cancer is shown in Table 3.

### 5.2. Salivary Liquid Biopsy

A clinical importance as a credible biofluid to detect systemic pathological conditions such as cancer has recently been attributed to the saliva, whose molecular components could reflect the systemic status of the body [166]. Therefore, the recent push for liquid biopsy together with understanding of salivary biomarkers provides a valuable source of information [167] to be used in precision medicine of the 3PM approach [8,168,169].

The combination of blood and saliva biomarkers could improve the detection of NSCLC. Salivary mRNA is considered a promising marker of liquid biopsy. Therefore, a novel biomarker panel consisting of combination of CTC levels in blood and mRNA markers in saliva (CCNI, EGFR, FGF19, FRS2, GREB1) could discriminate NSCLC from healthy controls [15]. Similarly, combined use of CEA in blood and salivary RNA biomarkers (GREB1, FRS2) could represent a novel liquid-based approach for NSCLC detection [170]. Moreover, a novel panel of five salivary miRNA has been identified as a promising tool for the diagnosis of CRC, supporting the role of salivary liquid biopsy as a novel approach to detect epigenetic alterations associated with cancer [11].

Salivary EV composition could be used to reflect local or systemic diseases to be utilized as a biomarker for oral as well as non-oral cancers. Changes in their composition in the case of non-oral cancers may be associated with their derivation from blood due to the vascularization of salivary glands or because of phenotypic changes in gland cells resulting from the stimulation by circulating tumor EVs [171]. As has been demonstrated in oral squamous cell carcinoma, salivary tumor-derived exosomes possess different morphological and molecular features when compared with healthy saliva samples, which highlights the possibility to detect malignant transformation in high-risk patients [172]. Interestingly, miRNA-1246 and -4644 in salivary exosomes have exerted potential use as candidate biomarkers for pancreatobiliary tract cancer [173]. Moreover, salivary exosomes harbor informative protein signature, which could be potentially used for the purpose of non-invasive detection of lung cancer [174]. A growing body of evidence highlights the role of cancer-derived salivary exosomes that originate from organelles and are transferred into saliva to be used as diagnostic biomarkers. However, the validation of salivary exosomes, the determination of molecular mechanisms regarding the interactions between salivary exosomes and distal tumors as well as establishment of rapid and sensitive technologies to purify and analyze salivary exosomes represent important future challenge of cancer research [175].

Considering the importance of human papilloma viral (HPV) DNA in saliva for HPV-associated oropharyngeal cancer (HPV-OPC), an acoustofluidic platform has been developed in order to isolate salivary exosomes, which are hypothesized to be packaged with HPV-associated biomarkers for salivary exosome-based liquid biopsy. Due to the packaging of HPV16 DNA sequences in HPV-associated oropharyngeal cancer in salivary exosomes, their isolation could improve HPV16 DNA detection [176]. New technology platforms that are based on the electrochemical detection of tumor-derived ctDNA in saliva are gaining importance due to advanced sensitivity and potential [166]. Electric field-induced release and measurement (EFIRM) is a novel platform allowing detection of ctDNA containing EGFR mutations directly from both plasma and saliva in early- and late-stage NSCLC patients. EFIRM liquid biopsy is an assay platform associated with circulating single-stranded DNA that is not targeted by any other extant platform [177].

Table 4 shows a detailed overview of current studies evaluating cancer-associated salivary biomarkers in liquid biopsy.

### 5.3. Cerebrospinal Fluid-Based Liquid Biopsy

Cerebrospinal fluid (CSF) could represent the best approach for minimally invasive diagnostics and disease monitoring of central nervous system (CNS) malignancies [178] in which plasma ctDNA is very low or absent [16]. In comparison with blood, the analysis of ctDNA in CSF has several advantages, including the following: lack of non-tumor cell-free DNA due to paucicellular nature of CSF; enriched ctDNA in CSF of patients with CNS-limited tumors; the fact that mutations in CSF ctDNA are most concordant with intracranial processes in metastatic CNS cancer; and the fac that CSF ctDNA could also uncover additional genetic aberrations reflecting tumor heterogeneity [179]. Importantly, CSF-based liquid biopsy has been demonstrated to be more sensitive than plasma-based in the management of patients with *ALK* (anaplastic lymphoma kinase)-rearranged NSCLC with leptomeningeal metastases [76]. Additionally, next-generation sequencing of CSF has been found to be superior to peripheral blood-based genetic testing at identifying uncommon *EGFR* mutation in NSCLC patients with leptomeningeal metastases [180].

Due to the altered metabolic pathways in cancer cells, the abnormal metabolic state of CNS cancer cells is hypothesized to be associated with abnormal levels of CSF metabolites, suggesting CSF metabolites as clinically useful tools for the management of CNS cancer patients. The analysis of CSF has revealed differences in the abundance of selected metabolites between control patients and patients with primary or metastatic CNS tumors [25]. Moreover, a combined analysis of plasma and CSF could be a useful tool in the management of HER2-positive breast cancer patients. CSF ctDNA reflects tumor burden variations and is potentially more sensitive and informative than traditional imaging. Therefore, CSF ctDNA could be used for monitoring of the progression and response to treatment of CNS lesions in the phenomenon of neurosystemic dissociation that is frequently observed during HER2-targeted therapy [181]. Moreover, CSF ctDNA could reveal mutation pattern in driver genes in brain metastases among NSCLC patients [182]. Therefore, CSF-based liquid biopsy represents an effective tool for personalized medicine and precision oncology in malignancies that are in some way associated with the nervous system [183,184].

A detailed description of mutation patterns or differences in metabolic profile as potential cancer biomarkers used in liquid biopsy performed on CFS is shown in Table 5.

### 5.4. Liquid Biopsy Based on Other Bio-Fluids

Within the current trends of 3PM medicine and the use of liquid biopsy as the precise medicine of various malignant diseases, there are many other sources of biomarkers that exert a promising potential in cancer management.

Pathological accumulation of fluid, which is also known as ascites, is related to various pathologies including cancer [185]. Malignant ascites contain tumor cells, fibroblasts, mesothelial cells, and inflammatory cells that produce various cytokines leading to the formation of tumor microenvironment in malignant ascites [30]. Patients with malignant ascites have poor prognosis and short overall survival [185]. Advanced cancer cases frequently show involvement of central nervous system, pleural or peritoneal involvement [149]. Ascites-based liquid biopsy could be an important prognostic biomarker for the evaluation of cancer and its microenvironment due to its accessibility. Tumor-infiltrating lymphocytes can be useful in the prediction of prognosis and response to immune checkpoint inhibitors. Importantly, a large amount of CD4+ and CD8+ T cells demonstrate an exhausted phenotype within gastrointestinal malignant ascites, which is associated with significant clinical relevance as a prognostic and therapeutic target in advanced gastrointestinal cancer [30].

The use of supernatant of PE may be more effective in the liquid biopsy due to content of many components released by tumor cells [186]. The majority of lung cancer cases are classified as NSCLC, which is often associated with mutations in the driving gene *EGFR* [187]. Importantly, liquid biopsy using EV-derived DNA from the supernatant of PE has been demonstrated as a promising approach for EGFR genotyping in pulmonary adenocarcinoma patients [186]. Moreover, a study evaluating the detection of *EGFR* gene mutations from different biofluids, including PE, ascites, pericardial effusion, and CSF, has revealed a higher detection rate and sensitivity of tumor-specific mutations in biofluid-supernatant-free DNA in comparison with biofluid sediment tumor cells and plasma-free DNA samples in patients with lung cancer [187].

EV-based liquid biopsy for *EGFR* genotyping using bronchoalveolar lavage fluid obtained from tumor sites has been related to highly accurate diagnosis of lung cancer patients, which is associated with the content of double-stranded DNA in EVs that reflects the mutational status of parental cancer cells in NSCLC [153]. Furthermore, *TMPRSS4* methylation status in bronchoalveolar lavage and plasma samples could be also used as a promising biomarker to monitor surgically resected NSCLC patients [188]. In addition, Li et al. have recently introduced a dual-layer precise, efficient, rapid, flexible, easy-to-operate, controllable, and thin (PERFECT) filter system that allows rapid liquid biopsy of lung cancer by separation and detection of exfoliated cancer cells from bronchoalveolar lavage fluid [189].

An evaluation of miRNA of EVs isolated from peritoneal lavage, a proximal fluid in CRC patients, and ascites from surgical CRC and non-cancer patients revealed their potential importance as untapped source of biomarkers [38]. Similarly, EV-associated miRNAs of peritoneal lavage have been demonstrated to be also a source of significant biomarkers in endometrial cancer [190]. Additionally, mutational analysis in peritoneal lavage and blood from early endometrial cancer has been recently found to be feasible, and further studies could determine its potential role in identification of patients with worse prognosis [191].

Interestingly, Martignetti et al. published a proof of principle that brings new opportunities in the future screening and detection of early cancer, as well as its prevention in asymptomatic individuals using targeted liquid biopsy. The identification of two oncogenic *PTEN* mutations nearly one year before the occurrence of symptoms of endometrial cancer through molecular analysis of uterine lavage fluid led to the diagnosis of a single microscopic focus of cancer in an illustrative case of a 67-year-old female [192]. Moreover, Casas-Arozamena et al. have recently published an article highlighting the personalized strategy in endometrial cancer patients based on the potential application of different liquid biopsies (uterine aspirate and blood samples) to monitor tumors and identify targeted therapies with probability to be extended to other gynecologic tumors [10]. Apart from mutational analyses, the microvesicle proteomic profiling of utero-tubal lavage has demonstrated potential as a biomarker for early diagnosis of ovarian cancer, while this approach could overcame the challenge of mutation-based assays that are complicated by rarity of tumor DNA within non-mutated DNA [35].

In addition, CA-125 level has been observed to be significantly increased in serum, cervical, and vaginal secretions of patients with complex hyperplasia and endometrial cancer and, therefore, could be potentially used as a diagnostic biomarker of precancerous disease or endometrial cancer [193].

Earlier data described modified expression of several tear proteins in breast cancer patients when compared with control females, suggesting an important role of proteomic pattern of tear fluid in the diagnosis of breast cancer [150,194]. Inubushi et al., who have revealed significantly higher quantity of exosome markers in tears than in serum, have recently confirmed the importance of tear fluid, especially tear exosomes, in breast cancer management. Moreover, miRNAs (miRNA-21 and miRNA-200c) specific to breast cancer were highly expressed in tear exosomes of metastatic breast cancer patients when compared to healthy control [195]. In addition to tear fluid, molecular analysis of breast milk could lead to the identification of proteins valuable for early detection and accurate assessment of breast cancer [151]. Moreover, nipple aspirate fluid that is directly derived from the breast ductal system is suggested to provide at least additional but potentially more sensitive and specific information on breast cancer management, especially when compared with liquid biopsy evaluating miRNA biomarkers in blood [196]. Additionally, an evaluation of miRNA expression in breast ductal fluid obtained by ductal lavage has revealed its feasibility and potential usefulness for the detection of breast cancer and discrimination of tumor histological subtypes [154].

Furthermore, seminal fluid is another source of ctDNA that could be used as a valuable biomarker, especially in prostate cancer. Ponti et al. demonstrated different concentrations and fragment size of seminal ctDNA in prostate cancer patients, benign prostate hyperplasia and healthy controls. Therefore, the use of automated systems for high-throughput ctDNA quantification could improve the implementation of the approach in clinical settings that support its potential in prostate cancer screening programs [33,152].

Table 6 summarizes results of current studies evaluating the potential use of liquid biopsy to analyze various cancer-associated biomarkers in biofluids other than blood, urine or saliva.

## 6. Liquid Biopsy as a Tool for Screening and Early Cancer Diagnosis

**Figure 3:**
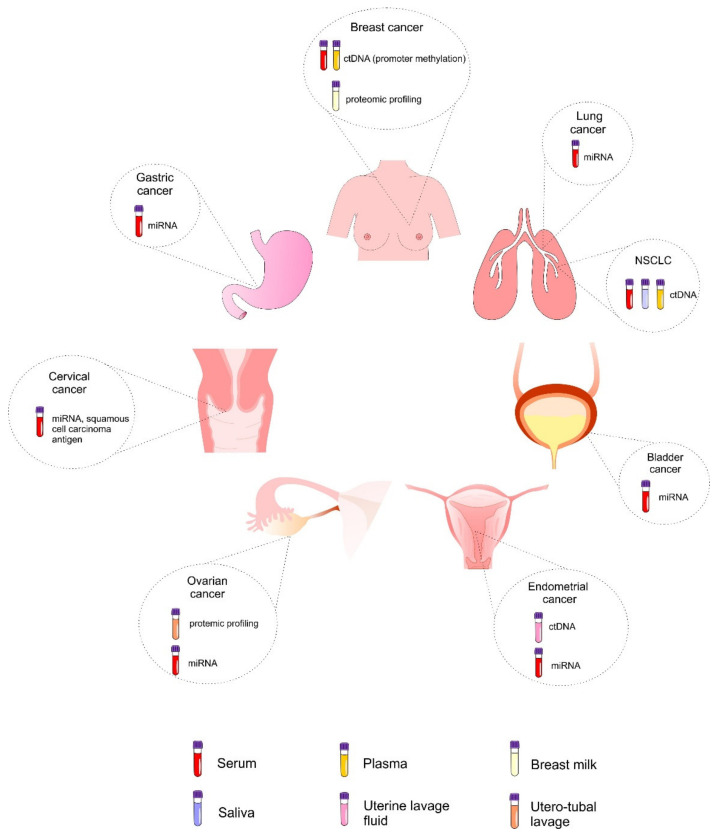
Biomarkers of liquid biopsy obtained from various samples of bio-fluids used for the early detection of selected cancer types. Abbreviations: ctDNA, circulating cell-free tumor DNA; miRNA, microRNA; NSCLC, non-small cell lung cancer.

The clinical importance of liquid biopsy is based on its applicability in all stages of cancer, allowing non-invasive and real-time monitoring of the disease [46]. The importance of screening is related to the detection of cancer at the early stage, that is, before the appearance of symptoms when the curative treatment is most likely to be successful. However, well-established biomarkers allowing early identification of cancer lack in most cancer types. Therefore, the clinical utilization of non-invasive methods such as liquid biopsy represent a promising approach for early cancer detection and improvements of survival rates of patients with various cancer types [197]. As discussed above, an evaluation of biomarkers from various types of liquid biopsy is associated with high applicability in the framework of early detection of various cancer types, such as lung [86,118,177], breast [112,151], bladder [120], gastric [95], cervical [98], endometrial [192], or ovarian cancer [35,121] (Figure 3). The application of liquid biopsy in cancer screening and early diagnosis represent the most promising aspects of its clinical utilization [46].

## 7. Concluding Remarks and 3PM-Related Expert Recommendations

Being systemic diseases, the absolute majority of cancers carry a multi-factorial character. Consequently, bio-fluids such as blood, urine, tear fluid, and saliva, among others, are instrumental for the prediction of cancer development and progression, reflecting systemic alterations in multi-omic biomarker patterns such as DNA methylation status, core of miRNAs and specific proteome [7,198,199]. Multi-parametric analysis of blood samples utilizing artificial intelligence such as (unsupervised) machine learning has been recommended for the prediction of cancer development, patient stratification for tailored therapy options, and disease monitoring [8,168]. The application of the multi-omic approach is considered to be crucial for cancer research and clinically relevant outcomes [200]. Although high specificity has been demonstrated for molecular patterns of individual tumors [201], individualized patient profiling including phenotyping and multi-level diagnostics is essential for personalized medical services in the clinical situation [202].

Although the application of 3PM concepts covers all stages of cancer management [203], the most cost-effective and patient-friendly approach remains targeted prevention, which is implementable to at least 30–50% of all cancer cases [1]. Liquid biopsy analysis is useful for innovative screening programs and targeting of individualized primary and secondary prevention [204,205,206].

Above all, the availability of various non-invasively or minimally invasively obtained samples (including the most commonly used blood, urine, saliva or various other biofluids such as lavage, tear fluid, breast milk or breast ductal fluid) of liquid biopsy offers a wide range of options that could be used as valuable biomarkers (such as ctDNA, miRNAs, CTCs, proteins, or metabolites) applied in precise cancer management in terms of predictive, preventive, and personalized requirements of modern medicine. The 21st century is significantly connected with personalized medicine. This novel mantra of healthcare is based on individualized patient profiling via multi-omic approaches. The identification of new biomarkers extracted from biofluids within non-invasive liquid biopsy demonstrates an appropriate strategy for monitoring response to therapy, recurrence of disease and thus improving the overall survival of patients with cancer. The constant development of technologies capable of detecting unique biomarkers brings new possibilities in the area of oncological research.

## Figures and Tables

**Table 1:** Detailed description of the advanced liquid biopsy technologies in cancer management.

Technology	Cancer Type	Liquid Biopsy Type	Biomarker	Reference
RT-PCR	CRC	Plasma	↑ Methylation of *SEPT9*	[48]
	CRC	Plasma	↓ miR-145, -195 ↑ miR-29, 92	[49]
	Gastric cancer	Plasma	↓ miR-195-5p	[50]
	Glioblastoma	CSF	↑ miR-21	[51]
	CNS malignancies	CSF	Differently expressed miR-771, -451, -223, -935, -125b	[52]
	UCC	Urine	gene expression signatures for UCC	[53]
	Bladder cancer	Urine	↓ miR-145, -200	[54]
	Bladder cancer	Urine	↑ UBE2C	[55]
	OSCC	Saliva	↓ miR-125, -200a	[56]
	EC	Saliva	↑ miR-10b, -21, -144 and -451	[57]
	EC	Saliva	↑ miR-21	[58]
	Lung adenocarcinoma	PE	↑ miR-195-5p, -182-5p, and -34a-5p	
dPCR	HNSCC	Plasma	Detection of *TP53* mutation	[61]
	Melanoma	Plasma	Detection of *BRAFV600E* mutation	[62]
	Metastatic adenocarcinoma	Plasma	Detection of *KRAS* mutation	[63]
	Central nervous system lymphomas	CSF	Detection of MYD88 p.(L265P)	[64]
	Glioma	CSF	Analysis of mutant *IDH1* mRNA	[67]
	Bladder cancer	Urine	Identification of *TERT* mutation	[65]
	NSCLC	Urine	Identification of *EGFR* mutation	[66]
	NSCLC	PE	Identification of *EGFR* mutation	[70]
NGS	NSCLC	Plasma	Detection of oncogenic drivers and resistance mechanisms	[74]
	EC	Plasma	Detection of somatic mutations associated with recurrence of disease	[83]
	LAC with LM	CSF	Detection of tumor associated mutations	[75]
	NSCLC with LM	CSF	Detection of targetable changes	[76]
	LC	PE	Identification of alterations in tumor genomics	[77]
MS	Multiple myeloma	Urine	Identification of urine biomarker	[80]
	Liver cancer	Serum	Analysis of total serum protein fingerprints	[81]
	ESCC	Plasma	Identification of plasma biomarkers	[82]

Explanatory notes: ↑ upregulated; ↓ downregulated. Abbreviations: CNS, central nervous system; CRC, colorectal cancer; CSF, cerebrospinal fluid; EC, esophageal cancer; ESCC, esophageal squamous cell carcinoma; HNSCC, head and neck squamous cell carcinoma; LAC, lung adenocarcinoma; LB, liquid biopsy; LC, lung cancer; LM, leptomeningeal metastases; NGS, next-generation sequencing; NSCLC, non-small cell lung carcinoma; OSCC, oral squamous cell carcinoma; PE, pleural effusion; UCC, bladder urothelial cell carcinoma.

**Table 2:** Blood-based liquid biopsy: potential roles of biomarkers for cancer management.

Biomarker	Cancer Type	Study Characteristic (Number of Patients)	Study Results	Reference
***Circulating tumor DNA***				
ctDNA (serum)	NSCLC	NSCLC patients (n = 60), COPD patients (n = 40) and healthy controls (n = 40)	Discrimination of normal individuals from COPD and NSCLC. ctDNA high level → short survival of NSCLC patients.	[86]
ctDNA (serum)	Advanced NSCLC	Patients rechallenged with gefitinib at progression after second-line chemotherapy (n = 61)	An identification of *EGFR*-mutant patients: those not carrying p.T790M variant with no other alternative treatment might benefit from TKI rechallenge.	[87]
Formalin-fixed, paraffin-embedded, metastatic tissue and corresponding ctDNA (serum)	Advanced breast cancer	Metastatic breast cancer patients (n = 66)	*PIK3CA* mutation tumor tissue and detectable *PIK3CA* mutations in serum ctDNA in 83% of cases. Correlation between changes in *PIK3CA* ctDNA level and treatment response.	[106]
HPV-specific E7 and L1 genes (ctDNA, plasma)	Cervical cancer	Cervical cancer patients (n = 138)	HPV E7 and L1 sequences detected in plasma ctDNA (61.6% of patients). High viral load: increased risk of recurrence and death at 5 years (univariate analysis).	[107]
Combined exosomal RNA and ctDNA (plasma)	NSCLC	Mutant EGFR NSCLC patients (n = 84)	Increased sensitivity for EGFR mutation detection, especially in patients with intrathoracic metastatic disease (low levels of ctDNA).	[108]
Methylation level in circulating cell-free DNA (serum, plasma)	Breast cancer	Serum test cohort (n = 103), serum validation cohort (n = 368), and plasma cohort (n = 125)	Serum test cohort: panel of SPAG6 and ITIH5 → 63% sensitivity of DCIS and 51% sensitivity for early invasive tumor detection at 80% specificity. The serum validation cohort: 50% sensitivity for DCIS detection (NKX2-6 and ITIH5).	[112]
Methylation level in circulating cell-free DNA (plasma)	Lung cancer	Tissue samples (n = 152), plasma samples (n = 129), and benign lesions of lung (n = 28)	Plasma samples: higher methylation of HOXA9 and RASSF1A in SCLC than in NSCLC.	[89]
Cell-free DNA sequencing informative methylation patterns (plasma)	More than 50 cancer types	Participants with (n = 8584) and without (n = 6670) cancer	Detection of more than 50 cancer types across stages.	[113]
***Circulating miRNAs in liquid biopsy***				
miRNA (serum)	Lung cancer	Preliminary marker selection (early-stage lung cancer n = 24 and healthy control n =24) and a validation phase (early-stage lung cancer n = 94, stage II to IV n = 48, and healthy control n = 111)	Potential of combination of miR-125a-5p, miR-25, and miR-126 in early detection of lung cancer.	[118]
miRNA (serum)	Prostate cancer	Prostate cancer patients (n = 809), negative prostate biopsies (n = 241), patients with other cancer types (n = 500), and healthy controls (n = 41)	Potential of combination of miR-17-3p and miR-1185-2-3p as a marker of prostate cancer diagnosis.	[119]
miRNA (serum)	Bladder cancer	Bladder cancer patients (n = 392), non-cancer samples (n = 100), and other cancer types (n = 480)	Set of 7 miRNAs (miR-6087, miR-1185-1-3p, miR-3960, miR-6724-5p, miR-1343-5p, miR-6831-5p and miR-4695-5p): discrimination of bladder cancer from non-cancer and other types of cancer.	[120]
miRNA (serum)	Breast, endometrial and ovarian cancer	Breast cancer (n = 31), endometrial cancer (n = 13), and ovarian cancer (n = 15) patients	miR-518b, -4719 and -6757-3p deregulated in breast cancer. miR-484/-23a diagnostic biomarker for endometrial and ovarian cancer.	[121]
***Extracellular vesicles in liquid biopsy***				
Circulating exosomes (serum)	ESCC	ESCC patients (n = 200)	Upregulated level of circulating exosomes in ESCC patients.	[91]
Circulating exosomal DNA (serum)	PDAC	PDAC patients (n = 48) and healthy subjects (n = 114)	Highlighting the role of circulating exosomal DNA in rapid identification of cancer driving mutations.	[92]
EV-miRNAs (serum)	mCRC	mCRC (n = 44) and healthy controls (n = 17)	Baseline miRNA-21 and -92a outperformed carcinoembryonic antigen levels in mCRC patients when compared to healthy controls. Higher levels of miRNA-92a and 222 in patients who died.	[93]
***Circulating tumor cells***				
CTCs	Prostate cancer patients	Prostate cancer patients with CTCs <5 (n = 511)	Increasing CTCs associated with worse overall survival of patients treated with chemotherapy of endocrine therapy.	[137]
	Metastatic breast cancer patients	Metastatic breast cancer patients receiving eribulin treatment (n = 21)	Determination of mesenchymal and epithelial CTCs for the prediction of survival.	
	SCLC	SCLC patients before pazopanib initiation (n = 56 patients), after one-cycle (n = 35), and on disease progression (n = 45)	Analysis of CTCs as biomarkers of treatment efficacy (pazopanib eliminates CTC subpopulations).	[139]
	EOC	EOC patients (n = 109)	Detection of CTCs and their pattern of gene expression could predict the likelihood of chemotherapy resistance and evaluate the prognosis of ovarian cancer patients.	[140]
	NSCLC	Advanced-stage NSCLC patients (n = 45)	Identification of CTCs through EGFR/HER3 expression →novel liquid biopsy approach.	[141]
	EOC	EOC patients (n = 10)	The feasibility and potential usefulness of chemosensitivity assay using liquid biopsy-derived CTCs in the prediction of response to therapy.	[142]
	Ewing sarcoma	Ewing sarcoma patients (n = 18) and healthy volunteers (n = 9)	Identification of CTCs by immunoseparation with CD99 antibody and magnetic microbeads → prognostic and predictive potential.	[143]
	CRC (draining venous blood)	CRC patients (n = 26) and healthy volunteers (n = 14)	New filtration and cytology-based automated platform for detection of CTCs → prognostic and predictive potential.	[144]
***Other biomarkers of blood-based liquid biopsy or their combinations***				
Soluble PD-L1 and PD-L2 (serum)	EOC	EOC patients (n = 83) and healthy controls (n = 29)	Soluble PD-L1 increased and PD-L2 decreased in EOC. Enhanced soluble PD-L1: residual tumor burden and reduced 5 year overall survival and progression-free survival. Reduced soluble PD-L2: platinum-resistance.	[145]
sCRT	Ovarian cancer	Ovarian cancer patients (n = 134) and healthy controls (n = 116)	Increased sCRT in ovarian cancer patients. sCRT predictor of poor prognosis and platinum resistance.	[94]
sGKN1	Gastric cancer	Advanced gastric cancer patients (n = 360), early gastric cancer patients (n = 140), and healthy controls (n = 200)	Increased sGKN1 in healthy subjects when compared with gastric cancer patients. Decreased sGKN1 in advanced gastric cancer when compared with early gastric cancer.	[95]
Serum proteins and miRNAs	Cervical cancer	Early-stage cervical cancer patients (n = 140) and healthy controls (n = 140). Independent cohort study (early-stage cervical cancer patients n = 60 and healthy controls n = 60)	Combination of SCC Ag degree and miRNA-29a, miRNA-25, and miRNA-486-5p levels as a marker of early-stage cervical cancer detection.	[98]
miRNA and fecal hemoglobin concentration (serum)	Colorectal carcinoma	CRC patients (n = 59), advanced adenomas (n = 74) and control subjects (n = 80)	Potential of a combination of 6 miRNAs (miR-15b-5p, miR-29a-3p, miR-335-5p, miR-18a-5p, miR-19a-3p and miR-19b-3p) and fecal hemoglobin concentration in the detection of advanced colorectal cancer in average risk individuals.	[146]
Histone modifications (serum)	Hepatocellular carcinoma	Hepatocellular carcinoma patients’ blood samples (n = 24) and healthy volunteers (n = 6)	Serum purified histones: comparable pattern of modifications like acetylation (H4K16Ac), methylation (H4K20Me3, H3K27Me3, H3K9Me3) and phosphorylation (γ-H2AX and H3S10P) to paired cancer tissues.	[96]
Histone modifications (plasma)	CRC	CRC patients (n = 63) and control subjects (n = 40)	Lower H3K27me3 and H4K20me3 in CRC patients when compared to healthy control.	[97]

Abbreviations: CD99, cluster of differentiation 99; COPD, chronic obstructive pulmonary disease; CRC, colorectal carcinoma; CTCs, circulating tumor cells; ctDNA, circulating cell-free tumor DNA; DCIS, ductal carcinoma in situ; EOC, epithelial ovarian cancer; ESCC, esophageal squamous cell carcinoma; HPV, human papilloma virus; mCRC, metastatic colorectal carcinoma; NSCLS, non-small cell lung cancer; PDAC, pancreatic ductal adenocarcinoma; SCC Ag, squamous cell carcinoma antigen; SCLC, small cell lung cancer; sCRT, serum calretinin; sGKN1, serum gastrokine 1.

**Table 3:** Potential role of cancer biomarkers in liquid biopsy performed on urine samples.

Biomarker	Cancer Type	Study Characteristics	Study Results	Reference
Tumor DNA (urine supernatant)	NMIBC	NMIBC patients (n = 216) and patients with bladder cancer undergoing radical cystectomy (n = 27)	An association between high levels of tumor DNA and later disease progression in NMIBC.	[158]
6-gene (*APC2*, *CDH1*, *FOXP1*, *LRRC3B*, *WNT7A* and *ZIC4*) promoter methylation (urine cell-free DNA)	Prostate cancer	Prostate cancer patients (n = 31) and control subjects (n = 33)	NGM increased monotonically from 0.27 in control subjects to 4.6 and 4.25 in patients with highly developed and T2/T3 stage metastatic prostate cancer, respectively.	[159]
ctDNA (plasma, urine)	mCRC	mCRC patients (n = 150)	Utilization of both plasma and urine cell-free DNA to address disease progression in CRC patients.	[160]
*EGFR* and *TP53* mutations (plasma, urine, sputum)	NSCLC	NSCLC patients (n = 50)	Increase in the detection of *EGFR* or *TP53* mutation with higher sensitivity by a combination of plasma, sputum and urine.	[161]
Lipids in urinary exosomes	Prostate cancer	Prostate cancer patients (n = 15) and healthy controls (n = 13)	Different levels of lipid species in the two groups.	[24]
miRNA	ccRCC	ccRCC patients (n = 75) and control subjects (n = 45)	Higher urinary cell-free miRNA-210 in ccRCC vs. control. Decreased urinary cell-free miRNA-210 in ccRCC patients a week after surgery.	[163]
miRNA (urine-derived exosomes)	Endometrial cancer	Endometrial cancer patients (n = 22) and symptomatic controls (n = 5)	The potential utilization of differential miRNA in exosomes as biomarker in diagnosis of endometrial cancer (hsa-miR-200c-3p as a candidate).	[165]
miRNAs	Bladder cancer	Identification of miRNA fingerprints: bladder cancer patients (n = 66) and control subjects (n = 48). Altered miRNAs validation: bladder cancer patients (n = 112) and control subjects (n = 65)	AUC (miR-30a-5p, let-7c-5p, miR-486-5p) altered in all bladder cancer subtypes → increased accuracy in the discrimination of cases and controls.	[164]

Abbreviations: AUC, area under the curve; ccRCC, clear cell renal cell carcinoma; CRC, colorectal carcinoma; ctDNA, circulating cell-free tumor DNA; mCRC, metastatic colorectal carcinoma; NGM, number of genes methylated; NMIBC, non-muscle-invasive bladder cancer; NSCLS, non-small cell lung cancer.

**Table 4:** Cancer biomarkers currently evaluated by saliva-based liquid biopsy.

Biomarker	Cancer Type	Study Characteristics	Study Results	Reference
mRNA (saliva) and blood CTCs	NSCLC	Discovery phase: NSCLC patients (n = 140) and healthy controls (n = 140). Validation phase: NSCLC patients (n = 60) and healthy controls (n = 60).	Panel of CTC level in blood and mRNA markers in saliva (CCNI, EGFR, FGF19, FRS2, GREB1): discrimination of NSCLC from healthy controls.	[15]
mRNA (saliva) and CEA (blood)	NSCLC	Discovery phase: NSCLC patients (n = 30) and healthy controls (n = 30). Prediction performance evaluation: NSCLC patients (n = 15) and healthy controls (n = 25).	Panel measuring CEA in blood and GREB1 and FRS2 levels in saliva could be used for the detection of NSCLC.	[170]
miRNAs (saliva)	CRC	Discovery phase (healthy controls n = 10 and CRC patients n = 14) and validation phase (healthy controls n = 37, CRC patients n = 51, and adenoma n = 19)	Panel of saliva-based miRNAs (miR-186-5p, miR-29a-3p, miR-29c-3p, miR-766-3p, and miR-491-5p) higher in CRC vs. control → detection of CRC.	[11]
miRNAs (salivary exosomes)	Pancreatobiliary tract cancer	Pancreatobiliary tract cancer (n = 12) and healthy controls (n = 13)	Relative expression ratios of miR-1246 and miR-4644 significantly higher in cancer group vs. control. The potential of miR-1246 and miR-4644 in salivary exosomes as candidate biomarkers.	[173]
Proteins (salivary exosomes)	Lung cancer	Lung cancer patients and normal subjects	The potential use of informative proteins in salivary EVs for detection of lung cancer.	[174]
Salivary exosomes			Isolation of salivary exosomes by the acoustofluidic (the fusion of acoustics and microfluidics) platform → potential in the detection of HPV-OPC.	[176]
ctDNA containing *EGFR* mutations (saliva, plasma)			Electric field-induced release and measurement → novel platform detecting ctDNA containing *EGFR* mutations directly from plasma and saliva in early- and late-stage NSCLC.	[177]

Abbreviations: CEA, carcinoembryonic antigen; CRC, colorectal carcinoma; CTCs, circulating tumor cells; HPV-OPC, HPV-associated oropharyngeal cancer; NSCLS, non-small cell lung cancer.

**Table 5:** The mutation patterns or metabolites differences as potential cancer biomarkers applied in CSF-based liquid biopsy.

Biomarker	Cancer Type	Study Characteristics	Study Results	Reference
CSF metabolites	Primary or metastatic central nervous system tumors	Patients without a history of cancer (n = 8) and with a variety of CNS tumor types (n = 23) (i.e., glioma IDH mutant, glioma IDH wildtype, metastatic lung cancer and metastatic breast cancer)	Differences in the abundance of 43 metabolites between CSF from control patients and the CSF of patients with primary or metastatic CNS tumors. Alterations in metabolic pathways (e.g., glycine, choline and methionine degradation, diphthamide biosynthesis and glycolysis pathways, among others) between IDH-mutant and IDH-wildtype gliomas. IDH-mutant gliomas: higher levels of D-2-hydroxyglutarate in CSF in comparison to patients with other tumor types or controls.	[25]
ctDNA (CSF, plasma)	HER2-positive breast cancer with brain metastases		CSF-derived ctDNA analysis: *TP53*, *PIK3CA* mutations and *ERBB2* and *cMYC* amplification. Post-treatment ctDNA analysis: decreased marker levels in plasma (consistent with extra-CNS disease control) and increased CSF (poor treatment benefit in the CNS).	[181]
ctDNA (blood, CSF)	NSCLC with brain metastasis	NSCLC patients with brain metastasis (n = 21)	Specific genetic mutation patterns in driver genes: *EGFR* mutations: 57.1% (in CSF ctDNA) and 23.8% (in peripheral blood ctDNA and in CTCs). *EGFR* mutations found in CSF of 81.8% patients with leptomeningeal metastases and 30% patients with brain parenchymal metastases. The status of *EGFR* and *TP53* mutations was consistent between CSF ctDNA and brain lesion tissue in all five patients.	[182]

Abbreviations: CNS, central nervous system; CSF, cerebrospinal fluid; CTCs, circulating tumor cells; ctDNA, circulating cell-free tumor DNA; HER2, human epidermal growth factor receptor 2; NSCLC, non-small cell lung cancer.

**Table 6:** Liquid biopsy based on other biofluids: the use of a broad spectrum of molecules as potential cancer biomarkers.

Biomarker	Cancer Type	Study Characteristics	Study Results	Reference
***Ascites-based liquid biopsy***				
Malignant ascites	Gastrointestinal cancer	Patients diagnosed with malignant ascites of gastrointestinal cancer (n = 27)	Large amount of CD4+ and CD8+ T cells: exhausted phenotype → significant clinical relevance as prognostic and therapeutic target.	[30]
***Pleural effusion***				
EGFR (pleural effusion, ascites, pericardial effusion and cerebrospinal fluid)	Lung adenocarcinoma patients	Lung adenocarcinoma patients (n = 20)	Higher detection rate sensitivity of tumor-specific EGFR mutations in biofluid-supernatant-free DNA in comparison with biofluid sediment tumor cells and plasma-free DNA samples.	[187]
***Bronchoalveolar lavage***				
EGFR (bronchoalveolar lavage fluid, EVs)	NSCLC	NSCLC patients (n = 137)	*EGFR* genotyping by bronchoalveolar lavage fluid obtained from tumor site: high accuracy of diagnosis.	[153]
*TMPRSS4* methylation (bronchoalveolar lavage and plasma)	NSCLC	Bronchoalveolar lavage: lung cancer patients (n = 79) and healthy controls (n = 26). Plasma: lung cancer patients (n = 89) and healthy controls (n = 25).	Monitoring of surgically resected NSCLC patients. *TMPRSS4* methylation status differentiates NSCLC and tumor-free subjects.	[188]
***Peritoneal lavage***				
EV-isolated miRNAs (peritoneal lavage, ascites)	CRC	CRC patients (n = 25) and control patients (n = 25)	Source of potential biomarkers for CRC diagnosis (miRNA-199b-5p, miRNA-150-5p, miRNA-29c-5p, miRNA-218-5p, miRNA-99a-3p, miRNA-383-5p, miRNA-199a-3p, miRNA-193a-5p, miRNA-10b-5p and miRNA-181c-5p).	[38]
EV-isolated miRNAs (peritoneal lavage, ascites)	Endometrial cancer	Endometrial cancer patients (n = 25) and control patients (n = 25)	Deregulated miRNAs in endometrial cancer (n = 114) miRNAs. miRNA-383-5p, miRNA-10b-5p, miRNA-34c-3p, miRNA-449b-5p, miRNA-34c-5p, miRNA-200b-3p, miRNA-2110, and miRNA-34b-3p highlighted as promising biomarkers.	[190]
*KRAS* and *PIK3CA* mutational analysis (peritoneal lavage, blood)	Endometrial cancer	Endometrial cancer patients (n = 50)	Approved feasibility of mutational analysis. Further studies needed to determine its use in the identification of patients with worse prognosis.	[191]
***Uterine/utero-tubular lavage***				
*PTEN* mutations (uterine lavage fluid)	Endometrial cancer	67-year-old asymptomatic female	Identification of two oncogenic *PTEN* mutations nearly one year before the occurrence of symptoms	[192]
Genomic profiling (uterine aspirate, blood)	Endometrial cancer	Endometrial cancer patients (n = 60)	Potential applicability of combined use of different liquid biopsy for personalized cancer management.	[10]
Microvesicle proteomic profiling (utero-tubal lavage)	Ovarian cancer	High-grade ovarian cancer patients (n = 49) and controls (n = 127)	9-protein classifier with 70% sensitivity and 76.2% specificity (identified all stage I lesions).	[35]
***Cervical and vaginal secretions***				
CA-125 (cervical and vaginal secretions, serum)	Endometrial cancer	Patients with polyps, hyperplasia or endometrial cancer (n = 97) and healthy subjects (n = 51)	Increased CA-125 in patients with complex hyperplasia and endometrial cancer.	[193]
***Tear fluid***				
Proteomic pattern	Breast cancer	Primary invasive breast carcinoma patients (n = 25) and healthy controls (n = 25)	Identified diagnostic protein biomarker to differentiate cancer patients from controls.	[150,194]
		Breast cancer patients (n = 50) and healthy controls (n = 50)		
Tear exosomes (miRNAs)	Breast cancer	Metastatic breast cancer patients (n = 5) and healthy controls (n = 8)	Higher quantity of exosome markers in tears than in serum. Highly expressed miRNA-21 and miRNA-200c in tear exosomes of metastatic breast cancer patients in comparison with controls.	[195]
***Breast milk***				
Proteins	Breast cancer	Ten milk samples from eight females	Identification of protein for early detection and accurate assessment of breast cancer.	[151]
***Breast ductal fluid***				
miRNAs	Unilateral breast cancer	Unilateral breast cancer patients (n = 22)	Feasibility of analyzing miRNAs. Discrimination of tumor histological subtypes. Discrimination of cancer and normal breast samples.	[154]
***Seminal fluid***				
ctDNA (seminal plasma)	Prostate cancer	Prostate cancer patients (n = 6) and healthy controls (n = 3)	Different concentrations and fragment size of seminal plasma ctDNA in prostate cancer patients, benign prostate hyperplasia and healthy controls.	[33,152]
		Prostate cancer patients (n = 30), benign prostate hyperplasia patients (n = 33), and healthy controls (n = 21)		

Abbreviations: CA-125, the cancer antigen 125; CRC, colorectal carcinoma; ctDNA, circulating cell-free tumor DNA; EVs, extracellular vesicles; NSCLC, non-small cell lung cancer.
